# UEFA’S financial fair play regulations: a good example of best practice governance by a sporting body?

**DOI:** 10.1007/s40318-021-00207-w

**Published:** 2022-01-17

**Authors:** Neil Dunbar, Thomas Middleton

**Affiliations:** College of Business, Law and Governance, James Cook University, Townsville, North Queensland Australia

**Keywords:** UEFA, Financial fair play regulations, Governance

## Abstract

The Union of European Football Associations (UEFA) is the body that governs European football. In 2010 it introduced its Financial Fair Play (FFP) Regulations with the main aim of bringing financial stability to European football. This article examines UEFA’s FFP Regulations from good governance and ‘best practice’ perspectives. It finds that the setting-up, implementation and monitoring of the FFP Regulations by UEFA generally adhere to ‘best practice’ principles. However, recent cases in the Court of Arbitration for Sport have revealed several areas where changes to the regulations would be appropriate. In 2021 UEFA made some changes to its Procedural rules governing its Club Financial Control Body, which address some of the outstanding issues but there are still a number of areas where changes would be beneficial as these would provide greater clarity and transparency to its operation and enforcement and thereby promote more timely and cost-effective regulation.

## Introduction

In March 2021 the Union des Associations Européennes de Football, or the Union of European Football Associations (widely known by its acronym UEFA), indicated its intentions to review its Financial Fair Play (FFP) Regulations due to the COVID-19 pandemic. UEFA’s director of research and financial stability, Andrea Traverso, stated that ‘COVID-19 has generated a revenue crisis and had a big impact on the liquidity of clubs’ adding that ‘the pandemic represents such an abrupt change that looking to the past is becoming purposeless.’^1^

UEFA’s FFP Regulations have been in place for approximately 10 years now and they initially received severe criticism from some commentators for being anti-competitive in nature.^2^ Legal proceedings were initiated twice, but both proceedings were subsequently discontinued.^3^ The FFP Regulations were suspended in 2020 due to the COVID-19 pandemic.^4^

The FFP Regulations have not been considered in the literature from an overall governance perspective although aspects of governance relating to them have been raised by some commentators on an ad hoc basis. It is proposed in this article to conduct a comprehensive analysis of whether the FFP Regulations embody good governance and best practice principles and to ascertain whether the regulations can be improved. This may be helpful to UEFA as it carries out its own review of its FFP Regulations and decides on the best approach to adopt to ensure the achievement of its objectives.

The initial part of this paper will set the scene looking at the importance of sport together with a consideration of the terms ‘governance’, ‘best practice’ and ‘regulation’ and their application in the sporting context. Attention will then turn to UEFA and what its FFP Regulations seek to do. The FFP Regulations will then be analysed in relation to good governance standards and best practices.

## Importance of sport

Sport is an area where governments have traditionally not become involved, working on the liberal principle that sporting bodies should be allowed to have freedom of choice over their decisions. However, with the considerable amount of money now flowing to sport through sponsorship, and television rights, and the large number of stakeholders currently involved and engaged in sport, it is not surprising that governments are showing greater interest in this area, particularly since they are often expected to provide significant funding. There are benefits for governments too. Governments appreciate that success in sport can bring prestige to a country and that economic benefits can also arise from hosting important sporting events, as the UK government found when hosting the Olympic Games in London in 2012.^5^ There is also the potential for governments to gain from the increase in taxation revenue associated with major/regular sporting events. The support of governments for sport may also increase its re-election prospects particularly where governments make donations for the construction of sporting facilities in marginal seats to gain popularity with voters. Apart from the financial and political issues associated with sport, there are also the social aspects to consider. Sport is also important to society because it increases public health and fitness. It has a public entertainment value, and brings society closer together, providing employment and career opportunities, as well as improving the facilities available to the community.

Due to the importance of sport in society there is a need for sporting bodies ‘to form partnerships, engage in dialogue and cooperate with governments.’^6^ There is also a need for sporting bodies to have ‘good governance procedures and practices’^7^ in place. If organisations do not have these procedures and practices in place, they ‘can expect their autonomy and self-regulatory practices to be curtailed.’^8^ Further, they will risk failing to achieve organisational and stakeholder objectives and may not survive in the long term.

## Governance

The word ‘governance’ comes from the Greek word *gubernatio*, which stems from the ancient Greek word *kybernao* meaning to steer or to guide.^9^ It has been used over many centuries. Cadbury refers to Chaucer’s use of the word and also quotes Cicero when he describes its meaning:

Governance is a word with a pedigree that dates back to Chaucer and in his day the word carried with it the connotation wise and responsible, which is appropriate. It means either the action of governing or the method of governing and it is in the latter sense it is used with reference to companies… A quotation which is worth bearing in mind in this context is: ‘He that governs sits quietly at the stern and scarce is seen to stir.’^10^

Governance involves a broad set of external and internal mechanisms and procedures that are designed to align the interests and objectives of the organisation with those of the various stakeholders to ensure the organisation’s: (a) compliance with the relevant laws and standards developed by professional and standard setting bodies; and (b) long-term survival.^11^

Sporting entities, like non-sporting business organisations, can be incorporated or alternatively configured in the form of partnerships or associations with some professional clubs operating on a profit basis and other amateur grassroots bodies running on a not-for-profit basis. Most sporting bodies operate with the intention that any profits made are ploughed back into their sport. By contrast, non-sporting business organisations focus on making large profits that are returned to investors. For example, directors of business corporations are expected to take calculated risks to maximise shareholder wealth.^12^ Despite these differences, sporting entities, however they are set up, are expected to meet the same legal and financial requirements as non-sporting businesses. Accordingly, sporting bodies, like non-sporting business entities, need to adopt good governance principles to ensure compliance and survival.

The Expert Group ‘Good Governance’ has defined good governance in sport as:

The framework and culture within which a sports body sets policy, delivers its strategic objectives, engages with stakeholders, monitors performance, evaluates and manages risk and reports to its constituents on its activities and progress including the delivery of effective, sustainable and proportionate sports policy and regulation.^13^

From this general definition it is apparent that a sporting body (like any non-sporting business entity), in order to adopt good governance practices, needs to produce effective and durable policies which meet its strategic objectives and take into account the interests of its stakeholders. It also needs to monitor its performance, manage risk, assess opportunities and be open and transparent in its operation so that stakeholders are kept fully aware of progress and what is happening.

## Best practice

There is, perhaps understandably, a degree of overlap between the terms ‘governance’ and ‘best practice’. However, there is a difference with governance being the broader, more overarching and general concept, whereas ‘best practice’ is best described as a subset of specific principles or procedures that apply depending on the nature of the organisation.

Governance can involve many different practices to ensure the organisation is achieving its own and aligned stakeholder goals and is more likely to survive in the long term. Consequently, there is a need to identify the ‘best’ governance ‘practices’ for each organisation, and for each area of activity, such as sporting activity, or commercial business activity. The literature indicates that ‘best practice’ principles are equally applicable to businesses and sporting bodies.^14^ Some of the more universal best governance practices that could apply to both sport and non-sporting business organisations include having:

- a competent board of directors/management team;
- strategies that are aligned with the goals of the organisation and stakeholders;
- procedures that promote compliance and accountability;
- systems/rules that promote a high level of ethics and integrity;
- an organisational plan that clearly defines roles and responsibilities; and
- procedures that identify and manage risk/threats as well as strategies for identifying and utilising/exploiting opportunities.

Vallabhhaneni provides the following definition of ‘best practice’

“Best practice” refers to processes, practices, and systems that are identified in top-performing public and private organisations and are widely recognized as improving the organisations’ performance and efficiency in specific areas. Successfully identifying and applying best practices can reduce expenses and can improve organisational efficiency.^15^

‘Best practice’ is now an established and acceptable approach to utilise in analysing, developing and reforming regulatory frameworks. Several academic writers, including Braithwaite and Drahos, have identified ‘best practice’ as being a main principle that is advantageous to use when examining global business regulatory regimes. They argue that businesses, investors and creditors and other stakeholders will move, or shift their capital, as the case may be, to jurisdictions where ‘world’s best practice’ has been adopted because they will have confidence that their investments and other interests are best protected in such a regulatory environment. ‘Best practice’ is not only concerned with principles about investment and finance but also ‘best practice’ in other areas, such as disciplinary procedures for sporting bodies, as this is the most timely and cost-effective way of producing fair and transparent outcomes in such matters.^16^

## Regulation

Regulation can be defined as ‘delegated legislation’ and ‘the act or process of controlling or directing by rule, restriction, principle, etc.’^17^ Baldwin, Cave and Lodge view regulation ‘as a specific set of commands—where regulation involves the promulgation of a binding set of rules to be applied by a body devoted to this purpose.’^18^

The need for regulation of business or sporting activity can be viewed as involving a regulatory pendulum. It is argued that when businesses or sporting organisations are performing successfully with a minimum number of collapses, scandals or abuses, the need for government prescriptive regulation is reduced and the government will favour a light-handed approach to regulation. It has been suggested that in this situation, the government will encourage self-regulatory initiatives by businesses or sporting organisations. Effective regulation or cost-effective compliance with the law may be achieved through self-regulation. Self-regulation involves having industry associations that help its members comply with the law or even exceed the legal requirements. If strong self-regulation measures are in place, this may promote compliance and eliminate or reduce the need for intervention by a government regulator.^19^

UEFA sits at the self-regulation end of the pendulum. It has responsibility for its own regulation, which involves the establishment of rules, their enforcement and the monitoring of the regulatory process.

Self-regulation is often favoured as a regulatory approach because it utilises expertise and is efficient.^20^ It is argued that the entity exercising self-regulation will have the best knowledge of its business and the area in which it operates to be in a good position to develop its own regulatory needs.^21^ From an efficiency perspective, it is contended that self-regulators are efficient because they have immediate access to those under their control and are able to easily obtain the information needed to establish and set the required standards and achieve the relevant objectives.^22^ Further, they have low monitoring and enforcement costs compared to government regulation.^23^

The main concern with self-regulation is the lack of enforcement action or power in cases of non-compliance. This problem arises because a body either fails to self-regulate or there is no statute available to provide sanctions for non-compliance. There are other concerns with self-regulation and these tend to involve the areas of mandate, accountability and fairness of procedure.^24^

These inherent features of the self-regulatory approach will be taken into account when assessing the FFP Regulations from good governance and ‘best practice’ perspectives.

## Best practice guides to regulation

There are several bodies that have prepared ‘best practice’ guides to regulation including the Organisation for Economic Co-operation and Development, and the Australian and New Zealand governments.^25^ These guides tend to follow the five criteria that Baldwin, Cave and Lodge suggest should be considered when assessing whether a regulation being adopted is good regulation.^26^ They are as follows:

- Is the action or regime supported by legislative authority?;
- Is there an appropriate scheme of accountability?;
- Are procedures fair, accessible, and open?;
- Is the regulator acting with sufficient expertise?; and
- Is the action or regime efficient?^27^

From a ‘best practice’ perspective, it is apparent that regulations must be legally enforceable and required to achieve the sporting body’s objectives, fully and properly planned with stakeholder consultation, implemented appropriately, monitored as to their effect and acceptance, and properly and justly enforced with natural justice or procedural fairness rules applying. The regulations must also meet the objectives of transparency and accountability in that each party subjected to the regulations must be treated similarly to other parties with the sporting body ensuring that this occurs.

Having considered the terms ‘governance’, ‘best practice’ and ‘regulation’, it is now appropriate to consider the UEFA organisation and then analyse whether it adopts good governance and best practice principles in a self-regulation framework.

## UEFA

UEFA was founded on 15 June 1954 in Basel, Switzerland.^28^ Currently, its offices are situated in Nyon and it employs over 450 staff.^29^ UEFA’s mission remains the same now as it was when it began in the early 1950s, namely ‘the fostering and development of unity and solidarity among the European football community’.^30^ However, ‘it has also become the guardian of football in Europe by working closely with its 55 member associations to promote, protect and nurture the sport at all levels, from the elite and its stars to the millions who play the game as a hobby’.^31^

## UEFA’S FFP regulations

In a move towards improving its governance of European football, UEFA introduced its Club Licensing Regulations (CLR) in 2004 requiring each national association to introduce a licensing system based on defined quality standards in the areas of sport, infrastructure, personnel, administration, law and finance. Football clubs wanting to compete in UEFA’s competitions were required to meet these standards.

Some clubs found it difficult to meet these new standards particularly in the area of finance. In 2009 UEFA’s research found that more than 50% of the 655 European football clubs had operated at a loss over the previous year. It also discovered that some clubs had large outstanding debts to creditors, including sums due to other clubs for transfer fees. It felt there was a distinct possibility of financial chaos with clubs becoming insolvent if attempts were not made to rectify the position.^32^

To address this issue UEFA introduced its FFP Regulations in 2010 with the aim of bringing financial stability to European football. UEFA sought to achieve this by requiring clubs to abide by a breakeven principle (that clubs spend no more than they earn) and to pay their debts in a timely manner.^33^ It also determined to monitor the FFP Regulations itself rather than allow the national associations to do this as continued to be the case with the CLR.^34^

## Analysis of UEFA’S FFP regulations

When considering ‘best practice’ it is important to take into account UEFA’s particular circumstances and recognise its role as the custodian of European football at all levels of the game and its mandate contained in its Statutes^35^ to represent the interests of its members, the national associations, together with the other stakeholders involved in European football.

In analysing UEFA’s FFP Regulations it is, therefore, proposed to consider the following areas:

- UEFA’s strategic objectives;
- Effective regulation;
- Appropriate planning and implementation, including stakeholder consultation;
- Monitoring the effect and acceptance of the FFP Regulations, including managing risk;
- The legality and necessity of the FFP Regulations;
- Transparency and accountability; and
- Enforcement of the FFP Regulations.

### UEFA’S strategic objectives

UEFA’s general objectives are contained in Article 2(1) of its *Statutes*. Objectives (a), (b) and (c) are general in nature but are important in that they emphasise the stewardship role of UEFA as promoting European football ‘in a spirit of peace, understanding and fair play’ as well as monitoring and controlling its development.^36^ Organising football competitions is a main task of UEFA and this is covered in objective (d).^37^ This task also provides UEFA with its income, so objective (d) also links in with objective (h) which requires UEFA to redistribute revenue generated from football in accordance with the principles of solidarity with the aim of supporting the grassroots of the game.^38^ Objectives (i), (j), and (k) require UEFA to promote unity among Member Associations and safeguard their interests together with ensuring that the interests of the different stakeholders in European football are taken into account.^39^ UEFA is also expected to act as a representative voice for the European football family.^40^ It can be seen from its general objectives that it is UEFA’s task to look after European football. It is suggested that UEFA has acted in accordance with its objectives when raising its concerns with the national associations about the financial viability of European football.

### Effective regulation

As stated above, UEFA’s aim was to bring financial stability to European football. To achieve this aim UEFA needed to find measures that could be applied fairly and evenly to all the clubs situated within the 55 different national associations, which UEFA administers. It would be inappropriate to have one rule applicable to clubs in some of the national associations and a different rule applying to clubs in other national associations. UEFA needed a consistent and simple approach to operate successfully throughout the whole of its jurisdiction which complies with the “rule of law” requiring that “like cases are treated alike.”^41^

UEFA’s main tool for bringing financial stability is the breakeven provision, which simply requires clubs to spend no more than they earn. This provision is hardly an unreasonable one and simply employs a ‘best practice’ standard and accepted business and accountancy principle to bring about the required change.^42^ It can operate across all 55 national associations. It is supported by a second measure of ensuring that club debts are paid in a timely manner. Once again this is a ‘best practice’ principle that is difficult to challenge from a business perspective and is hardly unreasonable. Both of the above provisions are ‘best practice’ in business because they reduce the need for debt finance, save on interest, and negate any potential creditor applications for winding-up on the grounds of insolvency. Thus it is suggested that UEFA has utilised effective and straight-forward regulation to achieve its aim.

Moreover, the effectiveness of the FFP Regulations in addressing solvency issues is demonstrable. Debt generally has tended to reduce substantially with UEFA revealing that the combined net debt of Europe’s top-division clubs has decreased from 65% of revenue in 2010 to 40% of revenue in 2018.^43^ Further, the impact of the FFP Regulations can be seen by contrasting the combined operating losses of Europe’s clubs in the 4 year period from 2009 to 2012 of €1 billion to the €3.6 billion operating profit for the 4 years from 2015 to 2018.^44^

### Appropriate planning and implementation including stakeholder consultation

The FFP Regulations have been carefully planned. UEFA appreciated that it was managing European football in 55 different countries. It needed a formula that would be straight-forward to operate within all those countries. The simple accounting procedures of spending no more than one earns and paying one’s debts in a timely fashion meet this requirement. Further UEFA, in its role of administering European football, was keen to extend its objectives of unity and consensus to its FFP Regulations. UEFA had a tenuous relationship with the wealthy clubs and was well-aware for some time of the threat of losing these clubs to a breakaway competition. UEFA recognised the importance of keeping these top clubs within the fold. This can be seen in the introduction of the breakeven principle as the principle was originally developed by the G-14 group^45^ which had discussed the idea of restricting wages to a maximum of 70% of a club’s turnover.^46^ Keeping the top clubs within the UEFA family is important for UEFA competitions.^47^ Losing the top clubs would have a disastrous impact on UEFA’s finances and would prevent it meeting its objectives to represent and assist all levels of football in Europe.^48^ The significant point here is that both restricting clubs to a percentage of turnover or the breakeven provision (only allowing them to spend what they earn) means that the wealthy clubs are still able to dominate the other clubs because they have more money to spend on player transfers and wages thus allowing them access to the better players. This may not promote competitive balance between the clubs but the purpose of the FFP Regulations was to ensure financial viability, not to promote competitive balance.

As part of its planning, UEFA proceeded to consult with its key stakeholders before finalising the nature of the FFP Regulations.^49^ Further, it ensured that it had their support before going ahead with them. UEFA also sought the support of the European Commission, the body carrying out the day-to-day administrative work on behalf of the European Union. In October 2014 the European Commission and UEFA signed a cooperation agreement acknowledging their progress in working together and providing a road map for joint work over the next 3 years.^50^ This agreement was renewed in 2018 for a further period of 3 years.^51^ UEFA’s efforts to obtain the approval of the European Commission were pivotal to its success with its FFP Regulations. If the European Commission had raised concerns relating to them, there would have been a greater likelihood of other parties also raising concerns.

It is interesting to note that only two legal proceedings have been commenced against UEFA in respect of its FFP Regulations. The first, in 2013, involved Daniel Striani, a player’s agent who was supported by some Manchester City and Paris St Germain (PSG) fans. He brought proceedings in the Belgium High Court but this case was dismissed in 2019 on a jurisdictional issue.^52^ The second involved a group of PSG supporters who commenced proceedings in the Paris High Court in 2015, but this matter appears to have been discontinued as no decision has been handed down by the Court. It is suggested that part of the reason for the lack of litigation against UEFA is due to the strenuous efforts that UEFA took to ensure general support by the stakeholders of the FFP Regulations before they were introduced. The lack of litigation about the FFP Regulations leads to lower costs of implementation of those regulations, which is an indicator of effective regulation and ‘best practice.’

UEFA ensured that the FFP Regulations would be implemented gradually, stating that the ‘phased implementation period will take place over 3 years, and the main component of the regulations—the ‘break-even’ requirement—will come into force for financial statements in the reporting period ending 2012…’^53^ and that this would ‘be assessed during the 2013–14 UEFA club competition season’.^54^ The then UEFA President Michel Platini said that ‘we have worked on the financial fair play concept hand-in-hand with the clubs, as our intention is not to punish them but to protect them’.^55^ It was also made clear that ‘during the implementation of the financial fair play rules, UEFA will continue to work together with clubs…’^56^ UEFA was determined to introduce the FFP Regulations fairly and over a reasonable time-frame and to give the clubs the opportunity to become familiar with them over an introductory period.

### Monitoring the effect and acceptance of the FFP Regulations including managing risk

Following reconsideration and reappraisal of the FFP Regulations, changes were announced in or about June 2015.^57^ These changes involved a relaxation of the breakeven requirement allowing clubs to make a short-term loss if they ‘can present a sustainable business plan and show that they will re-balance the books within a 3-year period’.^58^ The backdrop to the relaxing of the rules was the success of the FFP Regulations in reducing the combined losses of European football clubs by approximately 70% over the previous 3 year period.^59^ Platini, noted that ‘[w]e are just evolving from a period of austerity to one where we can offer more opportunities for sustainable growth and development’.^60^ He also added that ‘[t]he new regulations are an expansion and a strengthening of financial fair play’.^61^

UEFA probably realised it needed to provide an avenue for owners to invest in their clubs and if it did not, there was a reasonable chance the European Court of Justice could decide that the FFP Regulations were anti-competitive. However, UEFA was able to present the change as being a next stage in the process and to couch the voluntary agreement with financial safeguards to ensure it complied with its original and consistent aim of bringing financial stability to European football. Whatever UEFA’s actual reasoning for the introduction of the voluntary agreement it indicated that UEFA is an organisation which appreciated the need to monitor the effect and acceptance of its regulations and manage risk when required.

In addition, as mentioned in the introduction to this article, following the financial effects of the COVID-19 pandemic on European football, UEFA decided to conduct a further review of the FFP Regulations to ascertain whether they contain the most appropriate rules to be used in the changed climate. Andrea Traverso, director of research and financial stability, has indicated that ‘UEFA has begun consultation on how to reform FFP… saying he expected an “expedited and careful” process to be completed by the end of the year.’^62^ Currently the 2020 monitoring period of the breakeven provision has been postponed by 1 year and years 2020 and 2021 will be assessed together as one reporting period.^63^ UEFA probably had little alternative other than to take this approach. However, UEFA’s response to the difficult situation created by the pandemic supports the view that it is an organisation that is constantly overseeing its regulations to ascertain their effect and manage any associated risk. It also demonstrates UEFA’s continued effort to consult with its stakeholders.

### The legality and necessity of the FFP regulations

The voluntary agreement has probably also assisted UEFA in meeting its obligations to comply with European Union Competition Law (EUCL). The main stumbling block to the legality of the FFP Regulations is Article 101(1) of the *Treaty on the Functioning of the European Union.* This prohibits agreements which have ‘as their object or effect the prevention, restriction or distortion of competition within the internal market.’ Prima facie, it can be argued that forcing clubs to breakeven restricts competition. However, the voluntary agreement does provide a possible release of a club from the strict breakeven provision and this needs to be taken into account when considering the ancillary restraint exemption, which can apply to Article 101(1). This exemption can arise in situations where the agreement restrains trade, but the rule is held to be proportionate and aimed at achieving a legitimate and necessary objective of the organisation administering it.

The FFP Regulations satisfy these three elements. They are legitimate because European football did suffer from debt and overspending issues and UEFA’s aim in establishing the FFP Regulations was to protect the long-term viability of European football. The second element of necessity is also satisfied on the basis that ‘the imposition of timely payable payments and prudent budgetary management are likely also inherent in the pursuit of ensuring the sustainability and viability of European football clubs’.^64^ As to the third element of proportionality, the simple business approach of expecting clubs to pay their debts in a timely manner and to spend no more than they earn reflects a ‘best practice’ approach to ensuring the financial viability of the various clubs. The added possibility of clubs being able to take advantage of a voluntary agreement also supports the principles of proportionality and reasonableness.

### Transparency and accountability

The ‘best practice’ guides, discussed above, require that organisations, such as UEFA, strive to achieve transparency and accountability to maintain and promote public confidence in the integrity of its activities. UEFA needs to conduct matters transparently as there are a significant number of different stakeholders to consider and, being a large entity, accountability could be an issue if it is not consciously addressed. An ethical approach is necessary to achieve these objectives and is essential conduct in a cosmopolitan body where conflicting interests need to be managed honestly and carefully. UEFA’s general objectives, referred to earlier, cover this important area with objectives (e), (f) and (g) requiring UEFA to ensure European football functions and behaves ethically. It is apparent that UEFA has promoted transparency and accountability in its introduction and implementation of the FFP Regulations by ensuring that all stakeholders knew what the FFP Regulations entailed and what would be required of them, if anything. There was also a consultation period, which enabled stakeholders to have input into UEFA’s proposals, which promotes the transparency objective. Transparency and accountability will be further examined when looking at the enforcement of the FFP Regulations.

### Enforcement of the FFP Regulations

UEFA has set up the Club Financial Control Body (CFCB) as an independent body to enforce the FFP Regulations. The difficulty, however, is that there are always issues concerning an organisation’s transparency, accountability and legitimacy arising from questions and doubts about the independence of a subsidiary body (CFCB) that has been established by its parent organisation (UEFA) to administer the parent organisation’s rules. This is an inherent problem of the self-regulation approach, which was mentioned earlier. While UEFA established the CFCB to administer the FFP Regulations independently, UEFA is still responsible for the salaries of those CFCB personnel enforcing its rules. As a result, there will always be a concern about whether the body is truly independent.^65^ In these circumstances, it is crucial that transparency and openness are as manifest as possible in the conduct of the CFCB (the subsidiary and independent body), to ensure that it is clear that there is no UEFA influence in the CFCB process. It is also important that the disciplinary process used is as straight-forward as possible so that participants can easily understand how the disciplinary process will operate and how the rules will apply.

#### Structure of the CFCB

The CFCB was originally divided into the investigatory and the adjudicatory chambers. The words themselves are relatively clear in that ‘investigatory’ suggests investigating suspected contraventions of UEFA’s regulations and ‘adjudicatory’ suggests decision-making. But, in reality, the division was not as simple as this because the investigatory chamber could reach a decision to conclude a settlement agreement or dismiss a case. However, this was always subject to a possible review by the adjudicatory chamber if it did not agree with the CFCB chief investigator’s decision.

UEFA has addressed this clarity issue in its *Procedural rules governing the UEFA CFCB 2021* (PR 2021)^66^ with the two chambers being renamed the First Chamber and the Appeals Chamber. In this reformed structure the First Chamber acts as a court of first instance and the Appeals Chamber as an appellate body. The First Chamber now has the power to dispense a full complement of disciplinary sanctions as well as conclude voluntary agreements with clubs to ensure compliance with the breakeven requirements.^67^ The Appeals Chamber hears appeals from clubs which do not accept the First Chamber’s decision.

The new system makes the CFCB process much easier for participants to understand and follow. It creates a more logical hearing and appeals process and greater clarity as to what each Chamber does. It also provides an internal appeals process for participants, which provides for the CFCB to review its own first instance decisions. This is consistent with the disciplinary codes of other sporting bodies, like the International Federation of Association Football (FIFA), which operate a similar ‘two-tier judicatory mechanism.’^68^

#### Standard of proof

It is also appropriate that the procedural rules governing the CFCB are clear and transparent. However, the *Procedural rules governing the UEFA CFCB 2019* (PR 2019)^69^ and the PR 2021 do not deal with the standard of proof that is applicable to the CFCB proceedings. Express provisions dealing with the standard of proof would promote effective regulation as the parties no longer need to waste time and resources arguing about the applicable standard in such proceedings.

Article 25 of the PR 2021 states that ‘[i]n rendering its final decision, the adjudicatory chamber applies the UEFA Statutes, rules and regulations and, in addition, Swiss law’.^70^ Accordingly, Article 25 helps to ensure that CFCB decisions are based on relevant objective rules, rather than on any potential subjective pressures exerted by UEFA. However, from a transparency perspective more clarity would be helpful, and it would be sensible to extend the PR 2021 to cover the standard of proof, rather than to leave it to be covered by the ‘catch-all’ provision in Article 25.

Interestingly, the standard of proof is specifically mentioned in UEFA’s *Disciplinary Regulations* (DR)^71^ that apply to the Control, Ethics and Disciplinary Body, the body enforcing the Club Licensing Regulations. Article 24(2) states ‘[t]he standard of proof to be applied in UEFA disciplinary proceedings is the comfortable satisfaction of the competent disciplinary body’ and it is suggested that a similar standard should be set out in the PR 2021. ‘Comfortable satisfaction’ is the standard used by the Court of Arbitration for Sport (CAS) and it falls neatly between ‘the balance of probabilities’ used generally in civil matters and ‘beyond reasonable doubt’ used in criminal matters. The higher the standard of proof, the more protection there is for the clubs and the less likely it is that UEFA will achieve a successful regulatory outcome. It is suggested that ‘comfortable satisfaction’ provides a sensible standard or middle ground for sports cases because it assists to balance the interests of the clubs with the need for UEFA to obtain a successful regulatory outcome in particular cases.

#### Representation

The PR 2019 did not include any reference to clubs being represented at proceedings. This was changed in the PR 2021 with Article 21(1) providing that ‘a defendant or appellant may be represented by a person of choice.’ This is a positive improvement, but Article 21(1) should be amended to make it clear that it includes the right to legal counsel. The current wording could simply be extended to read ‘…by a person of choice *including legal counsel.*’ The use of lawyers for clubs promotes regulatory efficiency by reducing unwarranted objections during the process (compared to what happens with unrepresented litigants and litigants represented by non-legal personnel, who do not know the rules) and reduces the chances of unmeritorious appeals. This is because lawyers help ensure that the proper rules, procedures and processes are followed in the first place, in the First Chamber.

#### Valuation of sponsorship agreements and related party transactions

Another issue that requires consideration from the perspectives of transparency and openness is the valuation of sponsorship agreements. UEFA has experienced several difficulties with the valuation of sponsorship agreements, with some prominent clubs seeking ways to structure their agreements in order to have more money to purchase new players and still meet the breakeven requirement. Manchester City and PSG have had ongoing disputes with UEFA over these issues.^72^ Both clubs entered settlement agreements with UEFA in 2014, in which they were fined,^73^ but both clubs have been involved in further disputes since. Manchester City recently avoided a 2 year ban from UEFA competitions following an appeal to the CAS but was fined €10 million for not cooperating with the CFCB’s investigations.^74^ PSG escaped a potential penalty with CAS determining that UEFA’s decision to review PSG’s case must be reversed because it had failed to make its decision to review within the time limit of 10 days.^75^

Sims suggested that UEFA should introduce a number of reforms to assist with the valuation issue.^76^ He is of the opinion that harsher penalties should be imposed on repeat offenders, on the basis that, if they are only fined, it may be financially viable for a club to commit an offence and pay the fine, because the club is likely to be better off financially by taking this course of action.^77^ Sims also suggests the introduction of a new rule to prevent clubs from registering any player purchased in the financial year where a club is found to have breached the FFP Regulations.^78^ This would certainly make a club think carefully about deliberately breaching the FFP Regulations.^79^ Sims’ views are pertinent as it is important that repeat offenders receive harsher penalties to deter them and other clubs from continuing to offend. The topic of recidivism will be further considered in Sect. 9.7.7.

A further problem can arise in respect of transactions involving a ‘related party.’ Article 58(3) of the FFP Regulations states that ‘income and expenses from related parties must be adjusted to reflect the fair market value of any such transactions.’^80^ ‘Related party’ is defined as a person or entity that is related to the reporting entity.^81^ Some clubs seek to conceal equity contributions ‘as legitimate payments for services.’^82^ In other words, a club may use a ‘related party’ to obtain a larger payment for a service than the service provided actually warrants. By this means a club can gain an additional income, which, in effect, is an unapproved equity contribution.

Sims suggests that the definition of a ‘related party’ should be extended because UEFA’s definition is currently too narrow.^83^ He recommends that the definition should be similar to the definition used by the US Securities and Exchange Commission.^84^ He also suggests that the CFCB needs to adopt a broader interpretation of UEFA’s wording that two parties are related if they ‘are controlled, jointly controlled, or significantly influenced by the same government’.^85^ He points out how mystifying it was that the CFCB did not find that Etihad Airways was a ‘related party’ in its sponsorship of Manchester City.^86^ In the circumstances, Sims suggests that UEFA should consider strengthening its definition of ‘significant influence’ so that the CFCB has no option but to find in future cases that relationships like those between the owner of Manchester City and Etihad Airways, satisfy the definition of a ‘related party.’^87^ In this, Sims appears correct and UEFA should introduce a stronger definition of ‘significant influence’, especially given the narrow interpretation of the definition of a ‘related party’ that the CFCB has adopted to date, as seen with its Etihad Airways determination.

The valuation of a sponsorship agreement should also be carried out by UEFA in the first place, rather than allowing clubs to determine their own figures.^88^ This would remove any difference of opinion from the equation and would also make clubs more wary about entering into those transactions if they knew a valuation would be carried out by UEFA.^89^ Further, there would almost certainly be a delay in UEFA providing its valuation and this may inhibit clubs from pursuing related party sponsorships, since they are often seeking funds quickly.^90^ Sims supports this approach. Its main benefit is that UEFA could take control of the valuation process from the outset and thus be in a stronger position to deal with it.

UEFA should therefore review the troublesome area of valuations, particularly in relation to related party sponsorship agreements, where clubs may be seeking to secure a higher valuation than the agreement warrants to secure additional equity contributions. This would provide clearer and tougher regulations so clubs would think more carefully before trying to secure over-valued agreements, which could otherwise give them a clear advantage over other clubs that are honouring their breakeven requirements.

In addition, UEFA should improve its administrative support for the CFCB, which, as noted above, has lost two appeals cases in CAS as a result of exceeding time limits, due to administrative oversight and possibly inadequate staffing and/or heavy workloads. Additional funding to provide more staffing would probably assist the CFCB to meet its workload and satisfy its own procedural rules.^91^ It is noteworthy that UEFA changed Article 16 of PR 2019 to provide the adjudicatory chamber with a longer period of time to review decisions of the CFCB chief investigator.^92^ Of course, the recent change in Chamber structure of the CFCB means that the particular problem which arose in the earlier two cases may not occur under the new system provided any other possible issue mentioned above relating to adequate funding and staffing are adequately addressed.

#### Settlement agreements

Another area that requires consideration is the CFCB’s use of the settlement agreement which is often used by the CFCB to finalise an agreement with a club, with the aim of making that club comply with the FFP Regulations. The settlement agreement was originally concluded by the CFCB chief investigator but under Article 16 of the PR 2021 this responsibility has been transferred to the First Chamber to utilise ‘in circumstances that justify an effective, equitable and dissuasive resolution of the case.’^93^ The problem with Article 16 is that it does not give clear guidelines about when it can be used. For example, in the *International Skating Union* case^94^ the European Commission found that there were no ‘pre-established, clear and transparent criteria as to how the sanctions are to be applied’.^95^ This can create transparency and procedural fairness issues particularly with clubs that are not granted a settlement agreement.

Settlement agreements have been used in the majority of cases dealt with by the CFCB. However, a settlement agreement was not offered to AC Milan in its case. In the subsequent CAS hearing, *AC Milan v UEFA* (‘*AC Milan*’),^96^ the club’s legal representatives ‘argued that the regulatory framework for offering a settlement agreement was incompatible with EUCL, since the basis on which settlement might be offered is unclear and not set out in the Regulations’.^97^ It was submitted that compliance with EUCL required that ‘the conditions to be eligible for a settlement agreement are clearly known and explained to the clubs’.^98^ In essence, AC Milan argued that it was entitled to a settlement agreement and had been treated inequitably.^99^ Its breakeven deficit was no greater than that of Manchester City and PSG and they had received settlement agreements in 2014.^100^ CAS rejected this argument, viewing the settlement agreements and sanctions as being similar. It took the view that settlement agreements contain some form of sanction and that ‘the CFCB was entitled to choose one method of dealing with breaches over another as it deemed appropriate’.^101^ However, Nolan suggests there is a material difference between a settlement agreement, which is agreed by the parties, and a sanction that is unilaterally imposed. If this view was to be accepted in the future, it could be argued that the FFP Regulations breach EUCL due to UEFA’s unequal application of its sanctions.^102^ This is because unilaterally imposed sanctions may result in harsher penalties, compared to the sanctions contained in a settlement agreement, despite there being similar contraventions in both situations.

In the circumstances, it would be a proactive and pre-emptive move on UEFA’s part to remedy this situation. This would not be difficult, with Nolan stating that ‘UEFA need only amend Article 15 of the PR 2021 to properly define the circumstances in which a club may be offered a Settlement Agreement and ensure that these guidelines are applied in a fair and independent manner.’^103^ Even if the issue does not need to be resolved to meet EUCL requirements, good governance and ‘best practice’ principles require that UEFA has open and transparent rules that are equally applicable to all clubs.

#### Voluntary agreements

The procedure where clubs can apply for a voluntary agreement to breach the breakeven requirement for an agreed period of time, may benefit from a similar review to ensure its guidelines are clear and transparent. It should also be noted that the First Chamber of the CFCB is able to conclude a voluntary agreement under Article 14(6)(d).^104^ This is the first time that the CFCB has been given the jurisdiction to deal with voluntary agreements. Basic information about eligibility and process are provided in the FFP Regulations, but there are no published criteria about how the assessment of the club’s application for a voluntary agreement is conducted.^105^

Material about voluntary agreements appears sparse, with the only information obtained relating to AC Milan’s inability to secure one in 2017.^106^ Critical of UEFA’s handling of the AC Milan matter, Taormina stated, ‘AC Milan’s financial and ownership instability reveals UEFA’s lack of diligence of the financial risks that are inherent in the sale of European clubs to new buyers.’^107^ He puts forward two proposals to cover the situation of new clubs. First, he proposes that the FFP Regulations should be changed to allow clubs undergoing a change in ownership ‘to incur a higher deficit if it is completely covered by a direct injection of capital from the owners’.^108^ Second, he proposes that the FFP Regulations incorporate ‘a preliminary judgment process’ whereby potential new buyers are screened.^109^ These proposals should not be necessary and a transparent and equitably administered voluntary agreement system and the breakeven requirement should cover the situation satisfactorily. It might be appropriate for UEFA to offer advice to potential new owners if they request it but, as Taormina admits, ‘UEFA likely has no legal authority to *prevent* such a transaction.’^110^

The AC Milan example could be seen as a warning about the potential difficulties that could arise in respect of the voluntary agreement. It would be advisable for UEFA, from good governance and best practice perspectives, to take the necessary action to prevent this possibility from arising by providing criteria about how the assessment is conducted. To assist the clubs in their applications for a voluntary agreement, and to promote greater transparency and fairness, such criteria should be formulated and made available to the clubs.

#### Sanctions and recidivism

A further area which needs to be considered, from a transparency viewpoint, is that of sanctions and recidivism. In many respects, this is an extension of the issues concerning settlement agreements as they can be seen as falling within the field of sanctions. The sanction issue arose in *AC Milan*^111^ where the imposition of a 1-year ban from UEFA competitions was held to be not proportionate. The CAS panel held that ‘some important elements regarding the financial situation of the Club and the recent change in the Club’s ownership have not been properly assessed at the moment when the decision was rendered’.^112^ It referred the matter back to the CFCB to determine a new proportionate disciplinary measure. The CAS panel did not directly criticise UEFA’s sanctioning system *per se*, restricting its comments to the facts of the *AC Milan* case. However, it is apparent, as Bastianon says, that ‘UEFA does not apply clear and transparent criteria as to how its sanctions are to be applied.’^113^ Thus, using the same reasoning as was applied to settlement agreements, it is suggested that UEFA provide guidelines to the penalties which clubs can expect to receive for breaches of the FFP Regulations. This would not only promote personal and general deterrence but would also improve transparency. In addition, the guidelines would provide the CFCB with direction on potential punishments, which it could take into account when deciding on penalties.^114^

The PR 2019 did not refer to recidivism even though it is specifically covered in Article 25 of the DR.^115^ The PR 2021 now briefly deals with recidivism. One problem is that the new definition of recidivism is too narrow as it only applies to cases where the current contravention is similar to one that was committed by the same defendant within the previous 3 years and therefore does not cover recidivist conduct outside this period. Further, the PR 2021 merely states that recidivism is an ‘aggravating offence.’ There is no mention of the need for recidivist clubs to receive harsher penalties. It is suggested that the guidelines referred to above should specify that harsher penalties be imposed against recidivist contraveners. These guidelines should be contained within the PR 2021.

#### Tightening article 56 of the FFP Regulations: cooperation, obstruction and breach reporting

Transparency and accountability are a two-way street. It is incumbent on a regulator to provide the necessary information to the regulated to ensure they clearly know what is expected. Similarly, however, the regulated need to be transparent and cooperative in their responses to the regulator. This was not the case in the CAS proceedings between UEFA and Manchester City where the CAS panel held that Manchester City had breached Article 56 of the FFP Regulations by failing to cooperate with the CFCB’s investigations.^116^ The CAS panel indicated that the club had not only failed to cooperate, but had also obstructed UEFA’s investigations.^117^ As a result of this decision, UEFA has strengthened the rules by including Article 22(2) in its 2021 PR, which states that ‘[d]efendants/appellants must fully cooperate with the CFCB in respect of its requests and enquiries. If a defendant/appellant fails to fulfil its duty of cooperation, the CFCB may draw adverse inferences.’^118^

This provides greater enforcement and compliance powers than the CFCB previously had. It can be argued ‘that [t]his change could go a long way in ensuring that the CFCB has all the material it needs to reach an informed decision and creates an additional enforcement route against non-compliant clubs.’^119^ However, it can also be argued that Article 22(2) is not sufficient in that although it potentially enhances CFCB’s investigatory powers, it is not proactive in terms of securing compliance. A much stronger approach would be the introduction of an offence of obstructing the regulator.^120^ It is suggested that this would create greater encouragement to clubs to cooperate with CFCB’s investigations.

An alternative approach would be the placement of an obligation on clubs to report to UEFA if they breach, or are likely to breach, one of their obligations under the FFP Regulations. This type of proactive regulatory approach is used by the Financial Conduct Authority (FCA), which is the financial services industry regulator in the UK.^121^ Severe penalties could be imposed if it is later discovered that a club failed to make these breach reports. This suggested change is an example of ‘best practice’ as it promotes more timely and cost-effective regulation and compliance. It means that the regulator (UEFA/CFCB) would not always have to resort to its investigative powers and obtain information under compulsion, resulting in further delay and increased costs.

In addition, this latter approach could be linked to the inclusion in the proposed guidelines on sanctions (referred to above) of a provision that states that clubs may receive leniency or a lesser penalty if they cooperate with the CFCB’s investigations. The rationale for a lesser penalty is that cooperation saves UEFA/CFCB time and expense, and also helps to demonstrate the club’s contrition, remorse and acceptance of responsibility, which may suggest the club is more likely to rehabilitate and not repeat the conduct in the future

#### Introduction of new evidence

UEFA has also introduced reforms through its PR 2021 that prevent the introduction of new evidence to any appeal proceedings where that evidence was available at first instance. This appears to be in direct response to Manchester City’s introduction of new evidence to CAS in its recent proceedings. Article 34(3) of the PR 2021 provides that ‘CAS shall not take into consideration any substantial new facts or evidence that were available to or could reasonably have been discovered by the appellant and were not adduced by the latter before the CFCB.’^122^ Further, UEFA has also given its Appeals Chamber a discretion not to admit new evidence. Article 18(3) of the PR 2021 states that ‘[s]ubstantial new facts or evidence submitted by an appellant before the Appeals Chamber may be excluded by the latter, at its discretion’, if it was apparent that the evidence was either available or ‘could reasonably have been discovered by the appellant’ prior to the First Chamber’s decision.^123^

These changes to control the introduction of new evidence to UEFA’s/CFCB’s proceedings are to be commended and ‘may enhance the possibility of final resolution of proceedings at the CFCB without the need to go to CAS.’^124^ Further, from a practical perspective, they are likely to ensure that UEFA does not face a new evidence situation as it did in the CAS hearing with Manchester City.

These reforms mean that clubs will now be aware of the need to:

- clearly formulate the basis upon which they make their defence and to adhere to that basis;
- present their case completely at first instance; and
- not split the case by seeking to introduce so called ‘new’ evidence at an appeal where the clubs already had the evidence at first instance.

The reforms prevent clubs from engaging in tactical manoeuvres involving the introduction of so-called ‘new evidence’ at an appellate stage. Such tactics impose additional costs on the regulator and delay the regulatory outcome and compliance by way of unmeritorious appeals. The reforms will promote regulatory efficiency by ensuring that clubs complete their evidentiary case within a reasonable time at first instance.

#### Limitation period considerations

Another area of concern is the limitation period in which the CFCB must commence any proceedings. The period for commencing proceedings was set at 5 years^125^ and the CAS interpreted this in the Manchester City case to be 5 years from when the breach took place and that prosecution commenced when the matter was referred to the adjudicatory chamber for investigation. UEFA has reformed the limitation period in its PR 2021 so that the 5-year period commences on the day of the ‘breach’ and ends on the day of ‘the opening of proceedings’ which may provide a longer period for the First Chamber to carry out its investigations.^126^

It is suggested, however, that this reform does not overcome the problem that can arise if UEFA/CFCB does not become immediately aware of the breach. In the circumstances, UEFA needs to consider further reforming its limitation period so that time begins to run from when the CFCB knew or ought to have reasonably known of the breach. This would potentially give the CFCB more time to implement proceedings and would cover the situation where a club may seek to hide a breach. This type of wording is not uncommon in other legal/regulatory frameworks. It is used in cases of civil fraud, for instance, where the period of limitation does ‘not begin to run until the plaintiff has discovered the fraud…or could with reasonable diligence have discovered it’.^127^ Some regulatory bodies have a similar wording in their regulations. For instance, the FCA has 3 years from when it knew of the misconduct to commence proceedings.^128^ UEFA should amend its regulations so that the limitation period does not begin to run until the CFCB has knowledge of the club’s contraventions.

It is also suggested that UEFA enhances this regulation by including powers for the CFCB to obtain ‘relevant documents from clubs, their owners and their sponsors’, to conduct interviews and to audit accounts regularly.^129^ These additional powers would provide the CFCB with the ability to fully investigate a club’s activities, where appropriate. However, as mentioned earlier, UEFA would also have to ensure it had sufficient personnel to carry out investigations swiftly and efficiently. The added benefit of this stronger approach would be that clubs would have a greater incentive to comply with the regulations as they would appreciate that there would be a greater chance of a breach being discovered.

## Conclusion

UEFA’s decision to use the FFP Regulations to improve financial stability within European football reflects ‘best practice’ principles. The FFP Regulations were meticulously planned and implemented circumspectly. UEFA ensured it had stakeholder support for them prior to their introduction and was alert to the need to introduce the voluntary agreement which allows the possibility of investment by club owners in certain situations. It also promotes flexibility and avoids a rigid application of the breakeven principle and promotes greater stakeholder support for UEFA’s FFP Regulations.

Enforcement of the FFP Regulations has been placed in the hands of an independent body, the CFCB, with its procedural rules contained in the PR, which has appeared to run relatively smoothly, despite the concerns expressed previously about the independence of this body under the self-regulation model. It is suggested that as UEFA has been successful at eliminating major abuses, the European Commission has not seen the need to adopt an interventionist approach and has allowed UEFA to continue under the self-regulation model.

However, several regulatory issues have been identified in this article which it would be appropriate for UEFA to address. These include

- providing more clarity in relation to the criteria governing the use of settlement agreements and voluntary agreements;
- providing guidelines to sanctions including harsher penalties for recidivist conduct and lesser penalties where clubs cooperate;
- clarifying the standard of proof and the right to legal representation;
- reviewing the valuation of sponsorship agreements;
- redefining the definition of a ‘related party’;
- further strengthening the effect of Article 56 of the FFP Regulations by introducing an offence of obstructing the regulator (UEFA) and/or requiring clubs to report breaches or potential breaches of the FFP Regulations to UEFA; and
- amending the wording of the limitation period provision and increasing the CFCB’s powers to obtain evidential material.

These issues are not major failings in UEFA’s regulations but with the benefit of hindsight and what has occurred in recent cases before CAS, it does seem sensible for UEFA to make further adjustments in these areas to prevent potential regulatory difficulties in the future. These changes would produce more timely and cost-effective compliance and regulatory outcomes by reducing the need to resolve these issues on a litigious ‘case by case’ basis. It will also ensure that UEFA meets ‘good governance’ and ‘best practice’ objectives in relation to the enforcement and transparency of the FFP Regulations. The reforms suggested above would also promote greater accountability and stakeholder support and confidence in UEFA’s processes.

## Data Availability

Online.

## Footnotes

1 MacInnes (2021).

2 Flanagan (2013, p 163). See also Lindholm (2011, p 210), Serby (2016, p 50), Bodansky (2013, p 160), and Kaplan (2015, p 854).

3 This issue is further discussed in the Sect. 9.3 entitled ‘Appropriate Planning and Implementation including Stakeholder Consultation.’

4 This issue is further discussed in the Sect. 9.4 entitled ‘Monitoring the Effect and Acceptance of the FFP Regulations including Managing Risk.’

5 Pettiger (2012).

6 Expert Group “Good Governance” (2013, p 3).

7 Ibid.

8 Ibid.

9 Clarke (2007, p 1).

10 Cadbury (2002, p 1).

11 Rezaee and Riley (2010, p 125).

12 *ASIC v Mariner Corporation Ltd* (2015) 241 FCR 502; 106 ACSR 343; [2015] FCA 589 at [452]; and *ASIC v Avestra Asset Management Ltd (in liq)* (2017) 120 ACSR 247; [2017] FCA 497 at [214].

13 Expert Group “Good Governance” (n 6) 5.

14 Australian and New Zealand Sports Law Association (2021, p 48).

15 Vallabhaneni (2008, p 1).

16 Ibid.

17 LexisNexisButterworths (2015, p 371).

18 Baldwin et al. (2012, p 3).

19 Medcraft (2012, p 5).

20 Baldwin, Cave and Lodge (n 18) 139.

21 Ibid.

22 Ibid 140.

23 Ibid.

24 Ibid 139.

25 See Council on Regulatory Policy and Governance (2015). See also Australian Government (2015) and New Zealand Treasury (2015). Baldwin, Cave and Lodge (n 18) p 9.

26 Ibid 26.

27 Ibid 27.

28 UEFA (2020).

29 Ibid.

30 Ibid.

31 Ibid.

32 Dunbar (2015).

33 *UEFA Club Licensing and Financial Fair Play Regulations* 2018 arts 58-64 and 65-68. Two main areas are covered by the FFP Regulations: the breakeven requirement (Articles 58 to 64) and the enhanced no overdue payables rules (Articles 65 to 68).

34 Ibid art 57(1).

35 *UEFA Statutes* (2020 edition).

36 Ibid art 2(1)(a), 2(1)(b) and 2(1)(c).

37 Ibid art 2(1)(d).

38 Ibid art 2(1)(h).

39 Ibid art 2(1)(i), 2(1)(j) and 2(1)(k).

40 Ibid art 2(1)(l).

41 *Le v Secretary, Department of Education, Science and Training* (2006) 42 AAR 144; [2006] AATA 208 at [27]; *Soulemezis v Dudley (Holdings) Pty Ltd* (1987) 10 NSWLR 247 at [29]; and *Poidevin v ASIC* [2020] AATA 782 at [72]-[73].

42 Hughes (2019).

43 UEFA (2018a, b, p 126).

44 Ibid 110.

45 The G-14 was a group of the top and most wealthy European football clubs that existed between 1998 and 2008.

46 The Economist (2002).

47 Interestingly, only recently, in April 2021 12 of Europe’s leading clubs announced that they were forming a new mid-week competition, a Super League. The announcement was met with strong resistance from UEFA, supporters and commentators and a majority of the leading clubs decided to withdraw their support from the proposal immediately, making it an unviable proposition.

48 Taormina acknowledges that UEFA would be powerless to stop clubs from joining another competition and that ‘[l]ack of participation from Europe’s elite clubs would presumably cause a decrease in UEFA’s viewership and revenue and consequently, its dissolution’. See Taormina (2019, p 1292).

49 UEFA (2017a, b).

50 UEFA (2014).

51 UEFA (2018a, b).

52 Duval (2019).

53 UEFA (2011a, b, p 2).

54 Ibid.

55 UEFA (2010).

56 UEFA (2011a, b).

57 Homewood (2015).

58 Football Agents (2015). Note the current *UEFA Club Licensing and Financial Fair Play Regulations* (2018) Annex XII (A) (4) provides that a voluntary agreement can cover several reporting periods.

59 Associated Press 2015.

60 Homewood (n 57) 2.

61 Ibid.

62 MacInnes, (n 1) 1.

63 Ivanov (2020).

64 Taormina (2019, p 1305).

65 UEFA also provides the administration for the CFCB at UEFA headquarters. See *Procedural rules governing the UEFA Club Financial Control Body* (Edition 2019) art 11.

66 *Procedural rules governing the UEFA Club Financial Control Body* (Edition 2021) (PR 2021).

67 King, Shardlow-Wrest, Richmond and Jain, 2021.

68 Ibid 2.

69 *Procedural rules governing the UEFA Club Financial Control Body* (Edition 2019) (PR 2019).

70 PR 2021 art 25.

71 *UEFA Disciplinary Regulations* (Edition 2020) (DR).

72 Taormina sees the matter of related party sponsorship as being a major issue with the FPP and refers to the PSG and Manchester City cases as examples. See Taormina, (n 64) 1317–1320.

73 Weir (2014). See also CNN (2014).

74 Fox Sports (2020). See also McMahon (2020).

75 Court of Arbitration for Sport (2019). See also BBC Sport (2019).

76 Sims (2018, p 59).

77 Ibid 78. Sims’ suggestion appeared to have been taken on board, with Manchester City receiving a two year ban from UEFA’s competitions for its recent breach of the breakeven requirement. This was a second offence. However, CAS found that there had been no breach and the 2  year ban was withdrawn.

78 Ibid 79.

79 In Australian corporate law the courts indicate that the penalties should be set at a sufficiently high level to prevent them simply being regarded as a cost of doing business… *Re Make it Mine Finance Pty Ltd (No 2)* [2015] FCA 1255 at [45]; *ASIC v Commonwealth Bank of Australia* (2018) 128 ACSR 289; [2018] FCA 941 at [62]; *ACCC v ACM Group Ltd (No 3)* [2018] FCA 2059 at [25]; *ASIC v Mitchell (No 3)* [2020] FCA 1604 at [37]; and *ASIC v AGM Markets Pty Ltd (in liq) (No 4)* [2020] FCA 1499 at [33].

80 *UEFA Club Licensing and Financial Fair Play Regulations* (2018) art 58(3).

81 Ibid annex X(F)(1).

82 Sims (n 76) 71.

83 Ibid 81.

84 Ibid. In Australia the ‘related party’ definition is contained in ss 208–209 of the *Corporations Act 2001* (Cth) and is similar to the definition used by the US Securities and Exchange Commission.

85 Ibid.

86 Ibid.

87 Ibid. Manchester City’s owner is a member of the royal family of UAE and the owner of Etihad Airways is the UAE government.

88 Sims (n 76) 82.

89 Ibid 83.

90 Ibid.

91 The cases involved Galatasary and PSG. Both appeals took place in CAS in 2019 and UEFA failed because the CFCB had not made its determination within the allotted time period. See footnote 74 above. See also UEFA 2019.

92 PR 2019 art 16(1).

93 PR 2021 art 16(3)(b).

94 *Summary of Commission Decision (Case AT 40208 – International Skating Union’s Eligibility Rules)* [2018] OJ C 148, 9–12.

95 Nolan (2019, p 5).

96 CAS *AC Milan v *UEFA (2018a, b)/A/5808 (‘*AC Milan*’).

97 Nolan (n 95) 3.

98 Ibid.

99 *AC Milan* (n 96) 77(j).

100 Ibid.

101 Nolan (n 95) 3.

102 Ibid 4.

103 Ibid.

104 PR 2021 art 14(6)(d).

105 *UEFA Club Licensing and Financial Fair Play Regulations* (2018) Annex XII.

106 McMahon (2017). See also UEFA (2017a, b).

107 Taormina (n 64) 1322.

108 Ibid 1323.

109 Ibid.

110 Ibid. Approval for the purchase was presumably obtained from Serie A as this was the body directly affected by the change in ownership of the AC Milan club.

111 *AC Milan* (n 96).

112 Bastianon (2018, 6).

113 Ibid 8.

114 In Australia s 16A of the *Crimes Act 1914* (Cth) contains matters to which the court has to consider when imposing a sentence.

115 DR art 25(2).

116 Court of Arbitration for Sport (2020). See also *Manchester City FC v UEFA* CAS 2020/A/6785.

117 Ibid.

118 PR 2021 art 22(2).

119 King, Shardlow-Wrest, Richmond and Jain (n 57) 3.

120 The offence of obstructing a regulator is found in s 177 of the *Financial Services and Markets Act* (2000) (UK) and this may be utilised by the Financial Conduct Authority (FCA). Section 65 of the *Australian Securities and Investments Commission Act (*2001) (Cth) also contains an obstruction offence and this may be utilised by the Australian Securities and Investments Commission (ASIC).

121 The United Kingdom’s FCA supervises and monitors authorised firms’ compliance with the FCA's regulatory obligations. The FCA relies on regular notifications and regulatory reporting by firms to fulfil its supervisory role. See Financial Conduct Authority (2021). A similar obligation applies in Australia to financial services licensees, who must report breaches or likely breaches to the regulator (Australian Securities and Investments Commission) under section 912D of the *Corporations Act 2001* (Cth).

122 PR 2021 art 34(3).

123 PR 2021 art 18(3).

124 King, Shardlow-Wrest, Richmond and Jain (n 57) 3.

125 PR 2019 art 37.

126 PR 2021 art 37.

127 *Limitation Act 1980* (UK) s 32(1).

128 *Financial Services and Markets Act 2000* (UK) s 66(4).

129 Richmond et al. (2020).

## Code availability

To be agreed.
